# Supplementary material for: The evolution of parental care diversity in amphibians
Source: Nat Commun. 2019 Oct 17;10:4709. doi: 10.1038/s41467-019-12608-5 (PMC6797795; doi:10.1038/s41467-019-12608-5)
Supplement: Supplementary file 1 — Supplementary Information [file 41467_2019_12608_MOESM1_ESM.pdf]

Supplementary Information

The evolution of parental care diversity in amphibians

Andrew I. Furness and Isabella Capellini

This PDF includes:

Supplementary Note 1: Definitions of care behaviour and adaptations 2

Supplementary Tables 7

Supplementary Table 1 7

Supplementary Table 2 8

Supplementary Table 3 9

Supplementary Table 4 10

Supplementary Table 5 12

Supplementary Table 6 14

Supplementary Table 7 16

Supplementary Figures 17

Supplementary Figure 1 17

Supplementary Figure 2 18

Supplementary References 20

Supplementary Note 1: Definitions of care behaviours and adaptations

We defined Amphibian parental care as occurring post-fertilization[1]. As a result, we did not consider nest construction prior to or during mating as a form of parental care. We recorded all the parental care behaviours listed for each species in primary sources and reviews (please see Data Collection in Methods in the main text for information on the protocol we designed for scoring lack of care). Sources were found by searching Web of Science and Google Scholar using search terms such as 'amphibian', 'anura', 'caudata', 'gymnophiona' and 'parental care', among others. For some species parental care was indicated as facultative, or only observed for some clutches, or in only some populations. In these cases we followed the authors' interpretation regarding whether the species as a whole could be classed as exhibiting parental care. For some species there were conflicting reports on whether there was parental care or which sex provided care, while for others the sex providing parental care was unknown, or the validity of the original report had been questioned. We searched the literature for further information to resolve such contradictory information; however, when significant ambiguity still remained these species were eliminated from the dataset (see Data Collection in Methods in the main text). Specifically, we ran searches in Web of Science and Google Scholar, using the species' Latin name alone and in conjunction with relevant keywords such as 'parental care', 'reproduction', 'mating', and 'life history'. Altogether, we consulted 323 primary and secondary sources to assemble our dataset; the full dataset and list of sources are given in Supplementary Data 1 and 2 respectively.

We defined <u>egg attendance</u> as parent(s) remaining, full or part time, with the eggs at a fixed location[2, 3, 4, 5]. Egg attending parents have been observed to guard the eggs, actively defend the clutch against predators and conspecifics, protect the clutch against pathogens

by removing infected eggs, hydrate terrestrial developing eggs or aerate aquatic eggs that develop in oxygen poor pools[2, 3, 4, 5]. When studied, egg attendance has frequently been found to persist until the eggs hatch into tadpoles or into juveniles in direct developing species. We excluded from this definition any brief attendance that occurred only within the first few hours immediately following egg deposition, and instead considered this a component of reproductive behaviour at oviposition. For example, females of species in the family Centrolenidae have often been reported to stay with the eggs for a short time period lasting a few minutes up to several hours, and then permanently leave[6]. During this period, the egg jelly is hydrated by the female emptying her bladder while lying on top of the newly deposited eggs. In *Rhacophorus laterialis*, immediately following oviposition and lasting up to thirty minutes, the female alone constructs a leaf-nest surrounding embryos and foam, with no evidence of parental care after this period[7].

We defined egg brooding as the carrying of eggs on the parent's body (e.g. on the back or between the hindlegs) or within it (e.g. vocal sacs, stomach), ending with the release of tadpoles (no direct development) or froglets (direct development)[2, 3, 5]. While we did not discriminate between egg attendance and brooding species with or without direct development, we considered the duration of attendance or brooding by developmental stage in the analysis on the evolution of prolonged care (see Care Stage and Duration in Results and Discussion, and Data Collection in Methods, in the main text). Thus, in the analysis on the evolution of prolonged care, species with egg attendance or egg brooding and direct development are classed as exhibiting early and late, while those without direct development as having early care only (Supplementary Figure 1).

Tadpole attendance referred to parent(s) remaining, full or part-time, with aquatic or terrestrial larvae[2, 3, 4, 5]. Tadpole transport was defined as the transport of tadpoles on the parents' body from one location to another, after which the tadpoles are free-living[4]. In contrast, we classed as tadpole brooding cases where tadpoles complete most or all of their development in or on the body of the parent and are not free-living[4]. Specifically, in tadpole brooding species, the eggs are laid and develop in water or the terrestrial environment and, upon hatching, the tadpoles are taken up by the parent(s) and develop on or inside the carer's body[4].

We defined tadpole feeding as parental provisioning of tadpoles with fertilized or unfertilized eggs for the tadpoles to consume[2, 3, 4, 5]. Tadpole feeding could be easily identified when unfertilized trophic eggs are deposited by an unaccompanied female at regular intervals into tree holes or other confined areas which contained her tadpoles. However, there were several species in which tadpoles regularly or occasionally fed on fertilized eggs[8, 9] and it was not always clear whether these fertilized eggs were deposited as additional resources for the developing tadpoles, or whether the tadpoles were opportunistically eating fertilized eggs within a context of parent-offspring conflict. To score these species we accepted the primary authors' interpretation or used the consensus in the literature that considered tadpole feeding to be present when oophagy is required for metamorphosis; eggs are deposited multiple times or at regular intervals; there is mother-tadpole communication; or when tadpoles exhibit specialized morphology for oophagy. Ambiguous cases in which presence of tadpole feeding could not be determined were excluded.

Juvenile attendance was defined as parent(s) remaining, full or part time, with juveniles at a fixed location[5]. Juvenile transport referred to the transport of newly hatched froglets on the parent's body[4, 5]. There is variation amongst species in the length of time that froglets are carried (i.e. up to 9 days in *Liophryne schlaginhaufeni*[10]); we did not distinguish between transport and brooding at the juvenile stage, instead classing all these cases as juvenile transport with egg and/or tadpole brooding when relevant. Juvenile feeding (i.e. dermatophagy or skin-feeding) is a form of care found in some caecilians in which the female provisions juveniles with sloughed off skin[11, 12]. Lastly, viviparity, or live-bearing, refers to female gestation of offspring in the oviducts from fertilisation to birth[3, 4]. All viviparous species in our dataset reportedly can give birth to metamorphosed juveniles. Five species in the family Salamandridae exhibit intraspecific variation with some populations or individuals giving birth to larvae rather than juveniles. We classified these species as viviparous and giving birth to metamorphosed juveniles (i.e. both early and late care).

There were conflicting accounts regarding which sex exhibited a given parental care behaviour in some species of dendrobatids (families Aromobatidae and Dendrobatidae). Specifically, for egg attendance and tadpole transport we occasionally found statements in which one sex was indicated as the primary caregiver but sometimes, occasionally, rarely or infrequently, the other sex performed such duties. We scored each species on a case by case basis, generally favouring what had been the consensus scoring in the literature.

Tadpole transport, tadpole brooding, and/or tadpole feeding were recorded, but presence or absence of egg attendance (and if present, the sex of the carer) was not definitively reported in 39 species of the families Aromobatidae and Dendrobatidae.

Although Crump[2] indicated that all species in these families (except *C. stepheni*) probably had egg attendance, we were conservative and scored egg attendance as unknown; thus, because these 39 species had incomplete parental care data, they were then excluded from all analyses.

Five caecilian species exhibit juvenile feeding or viviparity but the presence or absence of egg attendance, juvenile attendance, or juvenile feeding could not be scored with confidence. As for egg attendance in the dendrobatids (above), these species with incomplete parental care data were excluded from all analyses.

The full dataset analysed for this study is available in Supplementary Data 1. All sources from which we have extracted the data on parental care in amphibians are listed in Supplementary Data 2.

Supplementary Tables

Supplementary Table 1. Sample sizes and character states (presence or absence) for *BayesTraits* RJ Multistate analyses of each parental care behaviour. In (a) all care forms by stage, in (b) care forms by function regardless of stage e.g. attendance at egg, tadpole or juvenile stage; transport at the tadpole or juvenile stage; brooding at the egg or tadpole stage; feeding at the tadpole or juvenile stage.

	Number of species		
**(a) Parental care behaviour**	**Absent (0)**	**Present (1)**	**Total**
Egg attendance	993	329	1322
Egg brooding	1275	47	1322
Tadpole attendance	1302	20	1322
Tadpole transport	1259	63	1322
Tadpole brooding	1315	7	1322
Tadpole feeding	1300	22	1322
Juvenile attendance	1311	11	1322
Juvenile transport	1310	12	1322
Juvenile feeding	1316	6	1322
Viviparity	1296	26	1322

	Number of species		
**(b) Parental care by function**	**Absent (0)**	**Present (1)**	**Total**
Attendance	992	330	1322
Transport	1252	70	1322
Brooding	1269	53	1322
Feeding	1294	28	1322
Viviparity	1296	26	1322

Supplementary Table 2. Transition rate estimates from *BayesTraits* RJ Multistate analyses for each parental care behaviour scored as present or absent. Sample sizes are reported in Supplementary Table 1a. MCMC analyses employed reversible jump (RJ) with an exponential prior with mean seeded from a uniform hyperprior ranging from 0 to 20. MCMC chains were run for 400 million iterations with a burnin of 500,000 and sampling every 200,000 iterations. MCMC runs for juvenile feeding exhibited chain mixing problems and therefore are discarded. Results for transition rate for the gain (**a**) and the loss (**b**) of parental care forms include the effective sample size (ESS), the mean, median and mode of the posterior distributions, the 95% highest posterior density (HPD) interval, and the percentage of models in the posterior in which a given parameter is estimated as equal to 0 (% zero).

**(a) Rate of gain** (q_{01})	**ESS**	**Mean**	**Median**	**Mode**	**95% HPD**	**% Zero**
Egg attendance	1787	0.366	0.363	0.348	[0.2936, 0.4520]	0.0
Egg brooding	2000	0.019	0.018	0.018	[0.0047, 0.0367]	0.0
Tadpole attendance	2000	0.059	0.057	0.051	[0.0231, 0.0947]	0.0
Tadpole transport	2000	0.026	0.025	0.024	[0.0081, 0.0457]	0.0
Tadpole brooding	2000	0.023	0.022	0.019	[0.0065, 0.0415]	0.0
Tadpole feeding	2000	0.070	0.065	0.056	[0.0178, 0.1372]	0.0
Juvenile attendance	1945	0.021	0.019	0.018	[0.0025, 0.0434]	0.0
Juvenile transport	2000	0.030	0.028	0.025	[0.0078, 0.0553]	0.0
Viviparity	2000	0.030	0.029	0.026	[0.0103, 0.0515]	0.0

**(b) Rate of loss** (q_{10})	**ESS**	**Mean**	**Median**	**Mode**	**95% HPD**	**% Zero**
Egg attendance	1796	0.366	0.363	0.348	[0.2936, 0.4516]	0.0
Egg brooding	2000	0.014	0.014	0.000	[0, 0.0337]	23.4
Tadpole attendance	1763	3.441	3.339	2.873	[1.5369, 5.6822]	0.0
Tadpole transport	2000	0.043	0.026	0.024	[0.0030, 0.1231]	0.0
Tadpole brooding	1154	0.049	0.019	0.022	[0, 0.0405]	19.7
Tadpole feeding	2000	9.290	9.262	9.212	[6.1738, 12.7253]	0.1
Juvenile attendance	2000	2.717	2.692	2.631	[1.0899, 5.2357]	0.3
Juvenile transport	2000	1.817	2.117	0.033	[0, 4.3427]	8.3
Viviparity	2000	0.047	0.027	0.027	[0, 0.0524]	15.7

Supplementary Table 3. Transition rate estimates from *BayesTraits* RJ Multistate analysis of parental care by function. Here we analyse care forms by function, regardless of the stage of development in which care is expressed (sample sizes in Supplementary Table 1b). For comparison we also report results from Supplementary Table 2 on viviparity. MCMC analyses employed reversible jump (RJ) with an exponential prior with mean seeded from a uniform hyperprior ranging from 0 to 20. MCMC chains were run for 400 million iterations with a burnin of 500,000 and sampling every 200,000 iterations. Results for transition rate for the gain (**a**) and the loss (**b**) of parental care functions include the effective sample size (ESS), the mean, median and mode of the posterior distributions, the 95% highest posterior density (HPD) interval, and the percentage of models in the posterior in which a given parameter is estimated as equal to 0 (% zero).

**(a) Rate of gain (q_{01})**	**ESS**	**Mean**	**Median**	**Mode**	**95% HPD**	**% Zero**
Attendance	1539	0.362	0.360	0.361	[0.2765, 0.4359]	0.0
Transport	2000	0.049	0.048	0.047	[0.0229, 0.0787]	0.0
Brooding	2000	0.034	0.033	0.030	[0.0128, 0.0548]	0.0
Feeding	2000	0.066	0.065	0.063	[0.0365, 0.1016]	0.0
Viviparity	2000	0.030	0.029	0.026	[0.0103, 0.0515]	0.0

**(b) Rate of loss (q_{10})**	**ESS**	**Mean**	**Median**	**Mode**	**95% HPD**	**% Zero**
Attendance	1461	0.362	0.360	0.358	[0.2765, 0.4364]	0.0
Transport	2000	0.059	0.048	0.047	[0.0230, 0.0839]	0.0
Brooding	2000	0.026	0.028	0.029	[0, 0.0514]	23.6
Feeding	1708	0.564	0.064	0.061	[0, 3.8515]	16.5
Viviparity	2000	0.047	0.027	0.027	[0, 0.0524]	15.7

Supplementary Table 4. Transition rate estimates from *BayesTraits* RJ Discrete Dependent analysis of early & late parental care. Analysis regardless of sex (Dependent vs Independent RJ Discrete models; BF=186.5) in (**a**); for males only (Dependent vs Independent RJ Discrete models, BF=133.8) in (**b**); and for females only (Dependent vs Independent RJ Discrete models, BF=183.7) in (**c**). MCMC analyses employed reversible jump (RJ) with an exponential prior with mean seeded from a uniform hyperprior ranging from 0 to 20. MCMC chains were run for 1 billion iterations with a burnin of 500,000 and sampling every 200,000 iterations. The columns report the transition rate between combinations of character states (Transition rate, where B=both early and late care, E=early care, L=late care, N=no care), the effective sample size (ESS), the mean, median and mode of the posterior distributions, the 95% highest posterior density (HPD) interval, and the percentage of models in the posterior in which a given parameter is estimated as equal to 0 (% zero). Sample sizes are as follows in (a): 258 species (B), 144 species (E), 8 species (L), and 912 species (N); in (b): 109 species (B), 76 species (E), 4 species (L), and 1133 species (N); in (c): 152 species (B), 82 species (E), 18 species (L), and 1070 species (N).

**(a) Early and late care regardless of sex**						
**Transition rate**	**ESS**	**Mean**	**Median**	**Mode**	**95% HPD**	**% Zero**
qNL	5000	0.285	0.284	0.280	[0.2246, 0.3451]	0.0
qNE	5000	0.285	0.284	0.280	[0.2246, 0.3451]	0.0
qLN	4662	12.652	11.836	10.851	[6.3923, 20.7947]	0.0
qLB	4771	12.555	11.783	10.889	[6.3923, 20.7947]	0.0
qEN	5000	0.287	0.284	0.280	[0.2246, 0.3481]	0.0
qEB	5000	0.287	0.284	0.276	[0.2216, 0.3456]	0.0
qBL	5000	0.286	0.284	0.280	[0.2246, 0.3480]	0.1
qBE	5000	0.285	0.284	0.280	[0.2246, 0.3453]	0.0

(b) Early and late care in males						
**Transition rate**	**ESS**	**Mean**	**Median**	**Mode**	**95% HPD**	**% Zero**
qNL	5000	0.177	0.176	0.175	[0.1254, 0.2309]	0.0
qNE	5000	0.177	0.176	0.175	[0.1254, 0.2309]	0.0
qLN	5000	24.890	21.770	15.004	[7.4633, 50.8383]	0.0
qLB	5000	24.770	21.676	14.976	[7.7155, 51.2247]	0.0
qEN	5000	0.612	0.217	0.180	[0.1211, 2.0927]	0.0
qEB	5000	0.387	0.198	0.177	[0.1255, 1.0476]	0.0
qBL	5000	0.381	0.197	0.177	[0.1254, 1.0754]	0.5
qBE	5000	0.119	0.157	0.174	[0, 0.2133]	32.8

(c) Early and late care in females						
**Transition rate**	**ESS**	**Mean**	**Median**	**Mode**	**95% HPD**	**% Zero**
qNL	1271	0.165	0.140	0.141	[0.0895, 0.2003]	0.0
qNE	4586	0.141	0.139	0.141	[0.0919, 0.1936]	0.0
qLN	1272	9.077	6.805	6.150	[3.8473, 15.4518]	0.0
qLB	2803	5.219	5.832	6.150	[0.7706, 8.6384]	0.0
qEN	4251	0.463	0.494	0.153	[0.0836, 0.9660]	0.6
qEB	3125	0.747	0.666	0.559	[0.1319, 1.5074]	0.0
qBL	3558	0.650	0.583	0.505	[0, 1.5036]	3.8
qBE	4682	0.311	0.167	0.145	[0.0815, 0.7905]	0.0

Supplementary Table 5. Transition rate estimates from *BayesTraits* RJ Discrete Dependent analysis on the correlated evolution of individual care forms across stages. Egg attendance without direct development and tadpole attendance (Dependent vs Independent RJ Discrete models; BF=53.8) in (**a**), egg attendance without direct development and tadpole transport (Dependent vs Independent RJ Discrete models, BF=23.0) in (**b**); egg attendance with direct development and juvenile care (Dependent vs Independent RJ Discrete models, BF=28.1) in (**c**). MCMC analyses employed reversible jump (RJ) with an exponential prior with mean seeded from a uniform hyperprior ranging from 0 to 20. MCMC chains were run for 400 million iterations with a burnin of 500,000 and sampling every 200,000 iterations. The columns report the transition rate between combinations of character states (Transition rate, where B=both early and late care, E=egg care, T/J=Tadpole or Juvenile care, N=no care), the effective sample size (ESS), the mean, median and mode of the posterior distributions, the 95% highest posterior density (HPD) interval, and the percentage of models in the posterior in which a given parameter is estimated as equal to 0 (% zero). Sample sizes are as follows in (a): 20 species (B), 191 species (E), 0 species (T), and 1111 species (N); in (b): 63 species (B), 148 species (E), 0 species (T), and 1111 species (N); in (c): 17 species (B), 101 species (E), 6 species (J), and 1198 species (N).

(a) Egg attendance without direct development and tadpole attendance

Transition rate	ESS	Mean	Median	Mode	95% HPD	% Zero
qNT	n/a	0.000	0.000	0.000	[0, 0]	100.0
qNE	1753	0.320	0.318	0.319	[0.2536, 0.3935]	0.0
qTN	2000	2.046	0.315	0.318	[0, 13.6115]	19.3
qTB	2000	0.627	0.317	0.318	[0, 0.6225]	11.4
qEN	2000	0.353	0.323	0.319	[0.2154, 0.5831]	0.0
qEB	2000	0.326	0.320	0.319	[0.2499, 0.3993]	0.0
qBT	732	0.253	0.304	0.318	[0, 0.3834]	26.7
qBE	2000	0.327	0.318	0.319	[0, 0.6587]	11.6

(b) Egg attendance without direct development and tadpole transport						
**Transition rate**	**ESS**	**Mean**	**Median**	**Mode**	**95% HPD**	**% Zero**
qNT	n/a	0.000	0.000	0.000	[0, 0]	100.0
qNE	2000	0.290	0.289	0.268	[0.2176, 0.3636]	0.0
qTN	2000	0.309	0.288	0.285	[0, 0.7374]	15.5
qTB	2000	0.323	0.292	0.279	[0, 0.7373]	8.7
qEN	2000	0.373	0.301	0.284	[0.2052, 0.9214]	0.0
qEB	2000	0.290	0.289	0.268	[0.2189, 0.3656]	0.0
qBT	2000	0.076	0.000	0.000	[0, 0.3214]	73.6
qBE	2000	0.329	0.295	0.285	[0.2085, 0.7499]	0.5

(c) Egg attendance with direct development and juvenile care						
**Transition rate**	**ESS**	**Mean**	**Median**	**Mode**	**95% HPD**	**% Zero**
qNJ	2000	0.010	0.000	0.000	[0, 0.0343]	61.1
qNE	2000	0.039	0.036	0.029	[0.0132, 0.0737]	0.0
qJN	2000	0.228	0.034	0.000	[0, 0.8877]	32.9
qJB	2000	0.293	0.041	0.000	[0, 0.9112]	29.4
qEN	2000	0.774	0.765	0.784	[0.5162, 1.0383]	0.0
qEB	2000	0.766	0.763	0.784	[0.5152, 1.0456]	0.0
qBJ	2000	0.645	0.738	0.784	[0, 0.9888]	8.3
qBE	1718	0.526	0.679	0.792	[0, 0.9861]	14.3

Supplementary Table 6. Transition rate estimates from *BayesTraits* RJ Discrete Dependent analysis of male & female parental care. Female and male parental care regardless of behaviour (Dependent vs Independent RJ Discrete models; BF=5.9) in (a); egg attendance (Dependent vs Independent RJ Discrete models; BF=18.6) in (b), and tadpole transport (Dependent vs Independent RJ Discrete models; BF=15.5) in (c). MCMC analyses employed reversible jump (RJ) with an exponential prior with mean seeded from a uniform hyperprior ranging from 0 to 20. MCMC chains were run for 400 million iterations with a burnin of 500,000 and sampling every 200,000 iterations. The columns report the transition rate between states (Transition rate, where B=biparental care, M=uniparental male care, F=uniparental female care, N=no care), the effective sample size (ESS), the mean, median and mode of the posterior distributions, the 95% highest posterior density (HPD) interval, and the percentage of models in the posterior in which a given parameter is estimated as equal to 0 (% zero). Sample sizes were as follows: (a) 31 species are biparental (B), 158 species with uniparental male care (M), 221 species with uniparental female care (F), and 912 species with no care (N); (b) for egg attendance 17 species are biparental, 164 male uniparental, 148 female uniparental, and 993 lack egg attendance; (c) for tadpole transport 3 species are biparental, 51 are male uniparental, 9 female uniparental and 1259 lack tadpole transport.

**(a) Male and female parental care (all behaviours)**						
**Transition rate**	**ESS**	**Mean**	**Median**	**Mode**	**95% HPD**	**% Zero**
qNF	2000	0.254	0.254	0.260	[0.1824, 0.3243]	0.0
qNM	2000	0.254	0.254	0.260	[0.1824, 0.3243]	0.0
qFN	2000	0.358	0.291	0.267	[0.1986, 0.6400]	0.0
qFB	2000	0.366	0.293	0.267	[0.1997, 0.6578]	0.0
qMN	2000	0.333	0.283	0.267	[0.1798, 0.5996]	0.0
qMB	2000	0.554	0.433	0.286	[0.2142, 1.3957]	0.0
qBF	2000	4.700	4.151	3.246	[1.5881, 8.9844]	0.0
qBM	2000	2.699	3.025	3.246	[0, 5.4452]	2.2

(b) Egg attendance						
**Transition rate**	**ESS**	**Mean**	**Median**	**Mode**	**95% HPD**	**% Zero**
qNF	1911	0.168	0.166	0.166	[0.1205, 0.2127]	0.0
qNM	1875	0.168	0.167	0.166	[0.1205, 0.2128]	0.0
qFN	2000	0.654	0.647	0.679	[0.4094, 0.9257]	0.0
qFB	1755	0.609	0.626	0.620	[0.1622, 0.8757]	0.0
qMN	1852	0.241	0.174	0.161	[0.1145, 0.6283]	0.2
qMB	2000	0.624	0.633	0.620	[0.1673, 0.8830]	0.0
qBF	2000	6.467	6.157	5.328	[3.3097, 10.7726]	0.0
qBM	1841	6.239	6.069	5.328	[3.0422, 11.3476]	0.0

(c) Tadpole transport						
**Transition rate**	**ESS**	**Mean**	**Median**	**Mode**	**95% HPD**	**% Zero**
qNF	1771	0.007	0.008	0.000	[0, 0.0170]	27.1
qNM	2000	0.013	0.011	0.010	[0.0028, 0.0308]	0.2
qFN	2000	0.185	0.009	0.000	[0, 0.7597]	36.0
qFB	2000	0.205	0.012	0.000	[0, 0.7579]	32.7
qMN	2000	0.502	0.490	0.473	[0, 0.8914]	0.9
qMB	2000	0.563	0.518	0.397	[0.1664, 1.0329]	0.0
qBF	2000	0.597	0.512	0.397	[0, 1.0474]	2.0
qBM	2000	0.327	0.020	0.000	[0, 0.9135]	22.7

Supplementary Table 7. Transition rate estimates from *BayesTraits* RJ Multistate analysis of type of biparental care. Sample sizes were as follows: 1291 species with no biparental care (N), 14 species with overlapping care (O), and 17 species with complementary care (C). MCMC analyses employed reversible jump (RJ) with an exponential prior with mean seeded from a uniform hyperprior ranging from 0 to 100. MCMC chains were run for 400 million iterations with a burnin of 500,000 and sampling every 200,000 iterations. The columns report the transition rate between states (Transition rate, where N=no biparental care, O=overlapping biparental care, C=complementary biparental care), the effective sample size (ESS), the mean, median and mode of the posterior distributions, the 95% highest posterior density (HPD) interval, and the percentage of models in the posterior in which a given parameter is estimated as equal to 0 (% zero).

Transition rate	ESS	Mean	Median	Mode	95% HPD	% Zero
qCN	1684	8.931	8.820	9.444	[5.8049, 12.2480]	0.0
qCO	1068	0.085	0.000	0.000	[0, 0.1337]	50.7
qNC	2000	0.068	0.072	0.078	[0, 0.1189]	15.0
qNO	1782	0.089	0.081	0.078	[0.0376, 0.1655]	0.1
qOC	1886	1.371	0.058	0.000	[0, 9.3151]	41.7
qON	2000	8.593	8.708	9.444	[5.0991, 12.7214]	2.1

Supplementary Figures

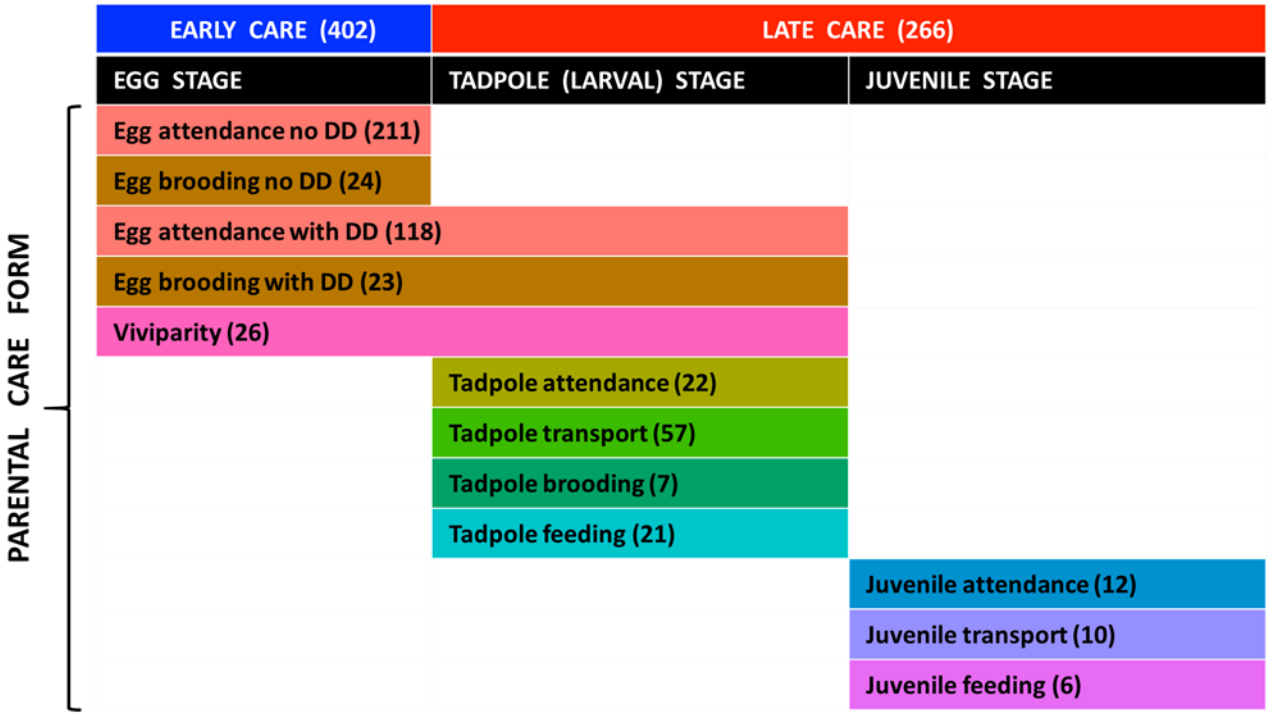

Supplementary Figure 1. Diagram summarizing the diversity of Amphibian parental care over the course of development. Numbers of species in each care category are reported, with colours matching those used in Figure 1 in the main text. We defined early care as care at the egg stage, thus species with egg attendance (with and without direct development, DD), egg brooding (with and without direct development, DD), or viviparity are scored as exhibiting early care. We defined late care as care at the larval or juvenile stage. Thus, late care included egg attendance and egg brooding in direct developing (DD) species, viviparity, tadpole attendance, tadpole transport, tadpole brooding, tadpole feeding, juvenile attendance, juvenile transport, and juvenile feeding. Note that, therefore, species with viviparity, egg attendance and brooding with direct development (DD), in which the offspring hatch as juveniles, are classified as having both early and late care (see Data Collection in Methods in the main text).

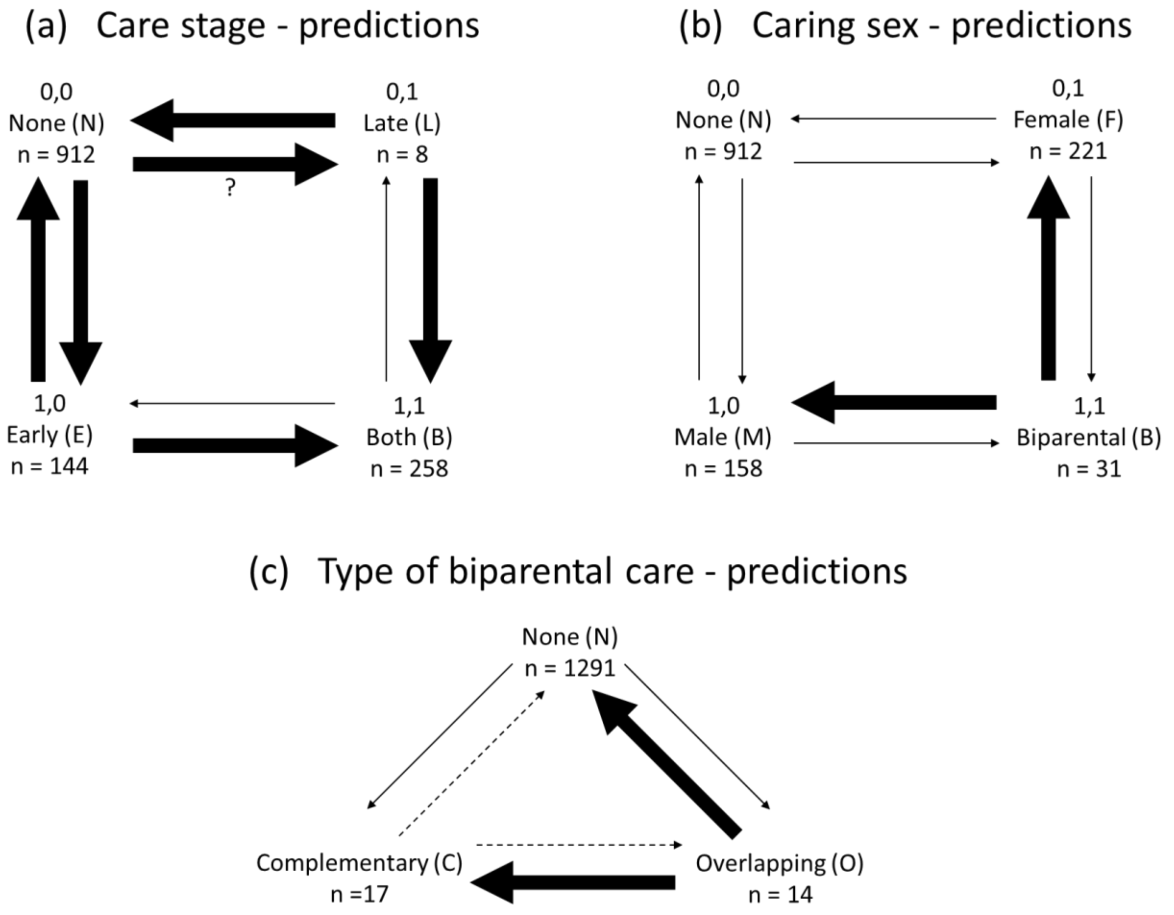

Supplementary Figure 2. Diagrams of predicted transition rate estimates for *BayesTraits* analyses of (a) prolonged care, (b) biparental care, and (c) type of biparental care. Across all figures, the thickness of the arrows indicates the predicted magnitude of the transition rates between combinations of character states, with thicker arrows meaning faster rates. In (a), we expect early care (E) to evolve from no care (N) and to act as a precursor or stepping stone towards the evolution of prolonged care (B), through the acquisition of care at late stages of offspring development. Also, we expect short term, early care to be easily lost back to no care. Conversely, if prolonged care is evolutionarily stable, it should be unlikely to revert back to early only care, and so the transition rate from early to prolonged care should be higher than those towards temporally limited care (early or late). There is currently no prediction in the literature for the evolution of late only care (L), a rare state in

Amphibians. Given that late only care is a form of temporally limited care, we assume it may evolve from no care but, given the lack of details in the literature, we cannot make more detailed predictions on the magnitude of the transition rates of this evolutionary pathway (indicated by the question mark). In (b), we expect uniparental male (M) and uniparental female care (F) to evolve (and be lost) at approximately equal rates from no care (N), and each to serve as a precursor or stepping stone towards the evolution of biparental care (B). However, once evolved theoretical models predict that biparental care is evolutionarily unstable and is quickly lost in favour of uniparental care. Thus, the magnitude of the transition rates leading to the evolution of biparental care from uniparental care are predicted to be smaller than those in the opposite direction. In (c), we expect complementary (C) and overlapping (O) biparental care to be gained at low rate from the absence of biparental care (N, i.e. either uniparental or no care). Furthermore, we expect that complementary biparental care (i.e. with division of labour) to be evolutionarily stable and exhibit a lower rate of loss (dashed line) relative to its rates of gain. In contrast, we expect overlapping biparental care, when the sexes perform the same care behaviours at the same stage of development, to be evolutionarily unstable and to exhibit higher rates of loss when compared to its rates of gain.

Supplementary References

1. Klug H, Alonzo SH, Bonsall MB. Theoretical foundations of parental care. In: *The evolution of parental care* (eds Royle NJ, Smiseth PT, Kölliker M). Oxford University Press (2012).
2. Crump ML. Parental care. In: *Amphibian biology* (ed Heatwole H). Surrey Beatty & Sons (1995).
3. Crump ML. Parental care among the Amphibia. In: *Parental Care: Evolution, Mechanisms, and Adaptive Significance* (eds Rosenblatt JS, Snowdon CT). Academic Press (1996).
4. Lehtinen RM, Nussbaum RA. Parental care: a phylogenetic perspective. In: *Reproductive biology and phylogeny of Anura* (ed Jamieson BG). Science Publishers, Inc. (2003).
5. Wells KD. *The ecology and behavior of Amphibians*. University of Chicago Press (2007).
6. Delia J, Bravo-Valencia L, Warkentin KM. Patterns of parental care in Neotropical glassfrogs: fieldwork alters hypotheses of sex-role evolution. *Journal of Evolutionary Biology* **30**, 898-914 (2017).
7. Biju SD. A novel nesting behaviour of a treefrog, *Rhacophorus lateralis* in the Western Ghats, India. *Current Science* **97**, 433-437 (2009).
8. Haddad CFB, Faivovich J, Garcia PCA. The specialized reproductive mode of the treefrog *Aplastodiscus perviridis* (Anura: Hylidae). *Amphibia-Reptilia* **26**, 87-92 (2005).
9. Schiesari L, Gordo M, Hödl W. Treeholes as calling, breeding, and developmental sites for the Amazonian canopy frog, *Phrynohyas resinifictrix* (Hylidae). *Copeia* **2003**, 263-272 (2003).
10. Bickford D. Animal behaviour: male parenting of New Guinea froglets. *Nature* **418**, 601-602 (2002).
11. Kupfer A, Maxwell E, Reinhard S, Kuehnel S. The evolution of parental investment in caecilian amphibians: a comparative approach. *Biological Journal of the Linnean Society* **119**, 4-14 (2016).
12. San Mauro D*, et al.* Life-history evolution and mitogenomic phylogeny of caecilian amphibians. *Molecular phylogenetics and evolution* **73**, 177-189 (2014).
