# Supplementary material for: The evolution of parental care diversity in amphibians
Source: Nat Commun. 2019 Oct 17;10:4709. doi: 10.1038/s41467-019-12608-5 (PMC6797795; doi:10.1038/s41467-019-12608-5)
Supplement: Supplementary file 3 — Reporting Summary [file 41467_2019_12608_MOESM3_ESM.pdf]

nature research

Corresponding author(s): Andrew Furness
Isabella Capellini

Last updated by author(s): Sep 1, 2019

Reporting Summary

Nature Research wishes to improve the reproducibility of the work that we publish. This form provides structure for consistency and transparency in reporting. For further information on Nature Research policies, see Authors & Referees and the Editorial Policy Checklist.

Statistics

For all statistical analyses, confirm that the following items are present in the figure legend, table legend, main text, or Methods section.

n/a	Confirmed	
☐	☒	The exact sample size (*n*) for each experimental group/condition, given as a discrete number and unit of measurement
☐	☒	A statement on whether measurements were taken from distinct samples or whether the same sample was measured repeatedly
☐	☒	The statistical test(s) used AND whether they are one- or two-sided<br>*Only common tests should be described solely by name; describe more complex techniques in the Methods section.*
☒	☐	A description of all covariates tested
☐	☒	A description of any assumptions or corrections, such as tests of normality and adjustment for multiple comparisons
☐	☒	A full description of the statistical parameters including central tendency (e.g. means) or other basic estimates (e.g. regression coefficient) AND variation (e.g. standard deviation) or associated estimates of uncertainty (e.g. confidence intervals)
☐	☒	For null hypothesis testing, the test statistic (e.g. *F*, *t*, *r*) with confidence intervals, effect sizes, degrees of freedom and *P* value noted<br>*Give P values as exact values whenever suitable.*
☐	☒	For Bayesian analysis, information on the choice of priors and Markov chain Monte Carlo settings
☒	☐	For hierarchical and complex designs, identification of the appropriate level for tests and full reporting of outcomes
☒	☐	Estimates of effect sizes (e.g. Cohen's *d*, Pearson's *r*), indicating how they were calculated

Our web collection on statistics for biologists contains articles on many of the points above.

Software and code

Policy information about availability of computer code

Data collection	No software was used to collect data.
Data analysis	Analyses were conducted using the program BayesTraits V3 and R version 3.4.2.

For manuscripts utilizing custom algorithms or software that are central to the research but not yet described in published literature, software must be made available to editors/reviewers. We strongly encourage code deposition in a community repository (e.g. GitHub). See the Nature Research guidelines for submitting code & software for further information.

Data

Policy information about availability of data

All manuscripts must include a data availability statement. This statement should provide the following information, where applicable:

- Accession codes, unique identifiers, or web links for publicly available datasets
- A list of figures that have associated raw data
- A description of any restrictions on data availability

The dataset compiled and analysed for this study is included in this published article and its supplementary information files (Supplementary Data 1 and 2).

Field-specific reporting

Please select the one below that is the best fit for your research. If you are not sure, read the appropriate sections before making your selection.

☐ Life sciences ☐ Behavioural & social sciences ☒ Ecological, evolutionary & environmental sciences

For a reference copy of the document with all sections, see nature.com/documents/nr-reporting-summary-flat.pdf

Ecological, evolutionary & environmental sciences study design

All studies must disclose on these points even when the disclosure is negative.

Study description	We assembled a species-level data set on Amphibian parental care and combine this with a phylogeny of Amphibians. We then perform phylogenetic comparative methods to test a number of hypotheses on the origin and maintenance of parental care in this diverse group.
Research sample	We assembled a dataset containing information on parental care presence or absence, type of care, sex of caregiver, and stage of development at which care occurs for 1,322 Amphibian species. This data was gathered from the literature.
Sampling strategy	All Amphibian species for which we were able to find data on presence or absence of parental care were included in the study.
Data collection	Data on Amphibian parental care was gathered from primary and secondary literature sources and recorded in an Excel spreadsheet.
Timing and spatial scale	The Amphibian parental care dataset was assembled between October 2017 and September 2018.
Data exclusions	Species for which there was significant ambiguity regarding parental care state (i.e. there were conflicting reports on whether there was parental care or which sex provided care, the sex providing parental care was unknown, or the validity of the original report had been questioned - and we were unable to resolve such contradictory information) were eliminated from the dataset, as were species with incomplete parental care information (i.e. only care type was known but not caregiver sex). The full dataset on which all analyses were performed is provided in Supplementary Data 1 and 2.
Reproducibility	All BayesTraits MCMC analyses were run in triplicate. Independent runs always produced qualitatively similar results.
Randomization	Our study was not experimental in nature and did not involve assigning individuals to different groups, so randomization is not relevant.
Blinding	Our study involved gathering existing data from the literature, and then analysing this data using phylogenetic comparative methods. Blinding is not relevant.

Did the study involve field work? ☐ Yes ☒ No

Reporting for specific materials, systems and methods

We require information from authors about some types of materials, experimental systems and methods used in many studies. Here, indicate whether each material, system or method listed is relevant to your study. If you are not sure if a list item applies to your research, read the appropriate section before selecting a response.

Materials & experimental systems

n/a	Involved in the study	
☒	☐	Antibodies
☒	☐	Eukaryotic cell lines
☒	☐	Palaeontology
☒	☐	Animals and other organisms
☒	☐	Human research participants
☒	☐	Clinical data

Methods

n/a	Involved in the study	
☒	☐	ChIP-seq
☒	☐	Flow cytometry
☒	☐	MRI-based neuroimaging
