# Supplementary material for: The evolution of parental care diversity in amphibians
Source: Nat Commun. 2019 Oct 17;10:4709. doi: 10.1038/s41467-019-12608-5 (PMC6797795; doi:10.1038/s41467-019-12608-5)
Supplement: Supplementary file 4 — Description of Additional Supplementary Files [file 41467_2019_12608_MOESM4_ESM.pdf]

Description of Additional Supplementary Files

File Name: Supplementary Data 1
Description: Amphibian parental care dataset.

File Name: Supplementary Data 2
Description: References used in construction of Amphibian parental care dataset.
